# Supplementary material for: Assessing the contribution of rare protein-coding germline variants to prostate cancer risk and severity in 37,184 cases
Source: Nat Commun. 2025 Feb 19;16:1779. doi: 10.1038/s41467-025-56944-1 (PMC11839991; doi:10.1038/s41467-025-56944-1)
Supplement: Supplementary file 1 — Supplementary Information [file 41467_2025_56944_MOESM1_ESM.pdf]

Supplementary Figures

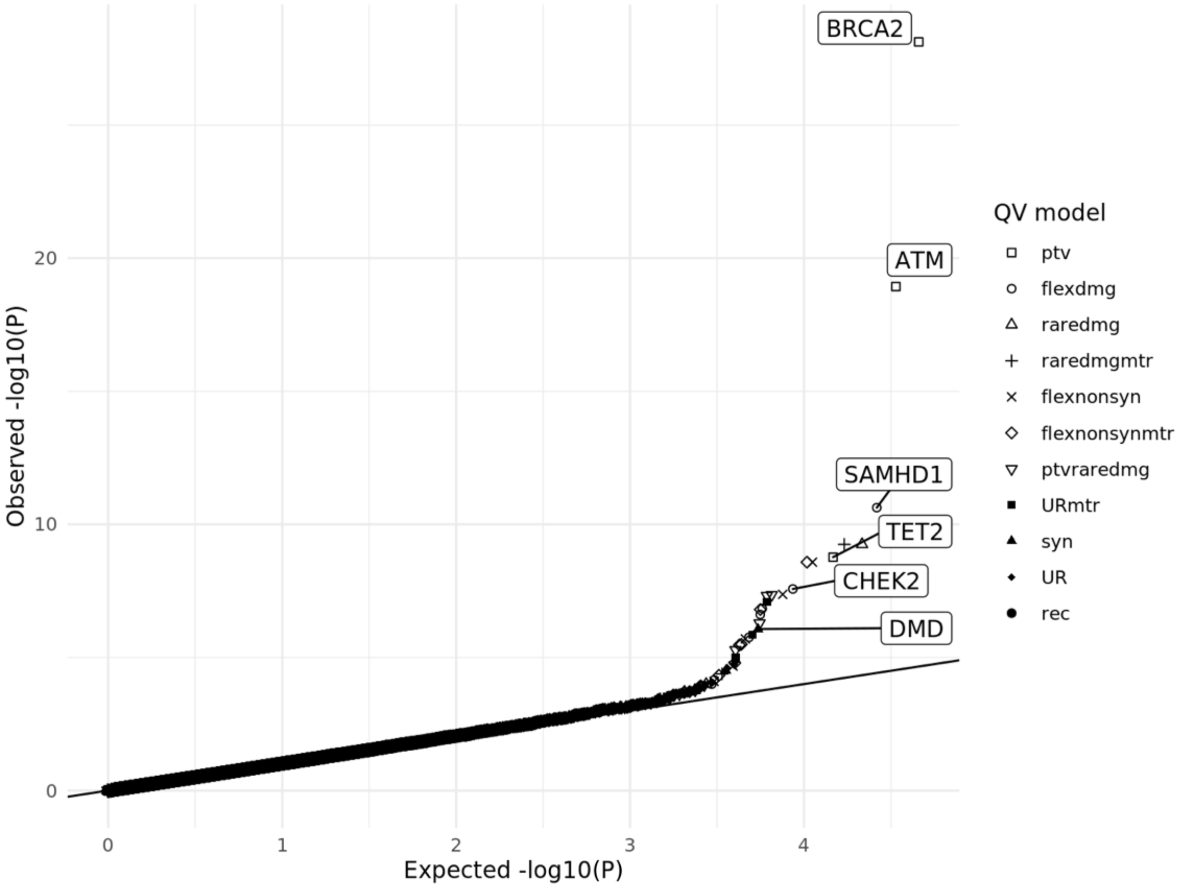

Supplementary Figure 1: QQ-plot for meta-analysis of gene-level association tests for all qualifying variant (QV) models for risk of developing prostate cancer (cases versus controls). Expected *P*-values on x-axis are generated from n-1 case-control permutation. *P*-values were determined from a Cochran–Mantel–Haenszel test across cohorts. Genes which reach the suggestive significance threshold ($P < 2.6\text{x}10^{-6}$) are labelled, and only the most significant QV model for each gene is labelled. All QV models defined in Supplementary Data 1.

BRCA2 (ptv)

Study	Odds Ratio (95% CI)	p-value
AZCT EAS	4.31 (0.67-27.87)	0.125
AZCT EUR	12.28 (5.47-27.53)	2.59e-09
MCPS	3.80 (1.01-14.33)	0.0483
UKB SAS	8.60 (1.19-62.13)	0.0327
NYBAZ EUR	6.17 (3.77-10.1)	1.91e-12
UKB EUR	2.67 (2.06-3.45)	5.74e-13
100kGP	1.99 (0.92-4.29)	0.08
Meta-Analysis	3.23 (2.65-3.90)	7.5e-29

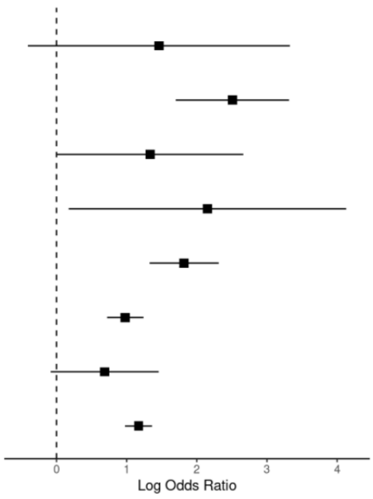

ATM (ptv)

Study	Odds Ratio (95% CI)	p-value
AZCT EUR	5.31 (2.46-11.45)	2.53e-05
MCPS	5.60 (1.83-17.15)	0.0026
UKB SAS	2.71 (0.37-19.65)	0.328
NYBAZ EUR	3.16 (1.81-5.52)	6.25e-05
UKB EUR	3.01 (2.25-4.01)	4.08e-13
Meta-Analysis	2.92 (2.34-3.63)	1.17e-19

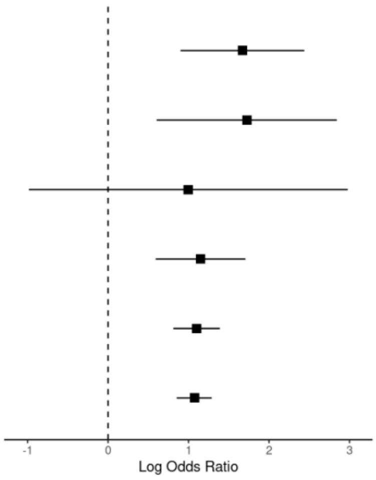

SAMHD1 (flexdmg)

Study	Odds Ratio (95% CI)	p-value
AZCT EAS	2.12 (0.34-13.36)	0.43
AZCT EUR	2.17 (1.03-4.59)	0.0414
MCPS	1.23 (0.62-2.44)	0.558
UKB SAS	2.49 (0.38-16.43)	0.35
NYBAZ EUR	1.23 (0.68-2.23)	0.5
UKB EUR	1.85 (1.43-2.39)	3.38e-06
UKB AFR	6.12 (1.37-27.23)	0.0173
100kGP	4.74 (2.65-8.49)	2.59e-07
Meta-Analysis	2.02 (1.65-2.45)	2.36e-11

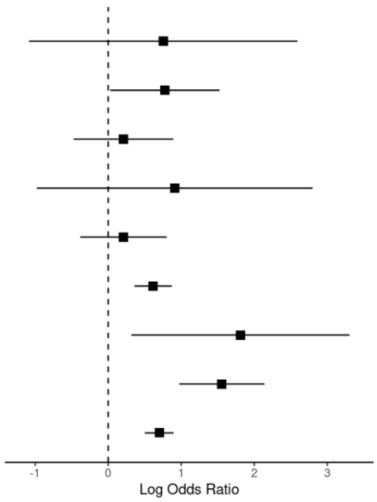

TET2 (ptv)

Study	Odds Ratio (95% CI)	p-value
AZCT EUR	1.31 (0.33-5.24)	0.714
MCPS	3.18 (0.40-25.07)	0.275
UKB SAS	14.95 (0.63-357.38)	0.0946
NYBAZ EUR	2.46 (0.81-7.49)	0.112
UKB EUR	2.68 (1.59-4.50)	0.000224
100kGP	16.28 (6.50-40.82)	5.63e-09
Meta-Analysis	3.31 (2.26-4.78)	1.71e-09

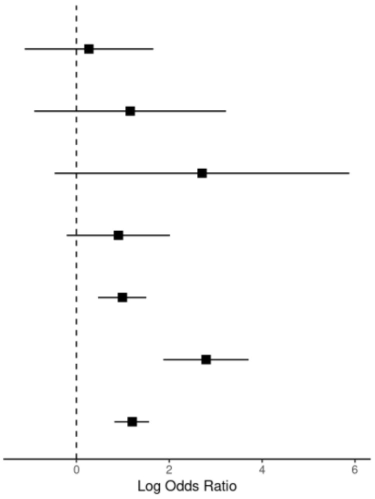

CHEK2 (flexdmg)

Study	Odds Ratio (95% CI)	p-value
AZCT EUR	1.23 (0.68-2.20)	0.503
MCPS	1.87 (0.76-4.60)	0.172
NYBAZ EUR	1.42 (0.88-2.32)	0.155
UKB EUR	1.91 (1.53-2.38)	1.38e-08
100kGP	1.39 (0.70-2.73)	0.353
Meta-Analysis	1.69 (1.41-2.01)	2.69e-08

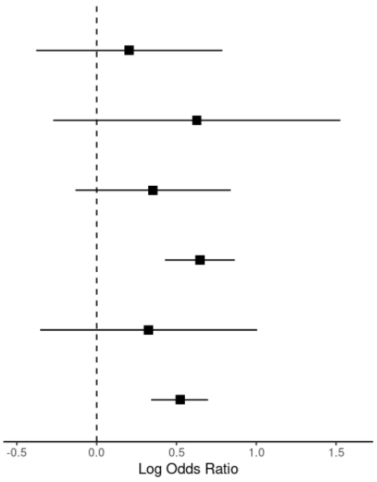

DMD (syn)

Study	Odds Ratio (95% CI)	p-value
AZCT EUR	0.19 (0.05-0.69)	0.0109
NYBAZ EUR	0.47 (0.15-1.43)	0.185
UKB EUR	0.55 (0.40-0.76)	0.000368
UKB AFR	0.46 (0.01-21.16)	0.707
100kGP	0.52 (0.13-2.11)	0.365
Meta-Analysis	0.50 (0.36-0.67)	8.6e-07

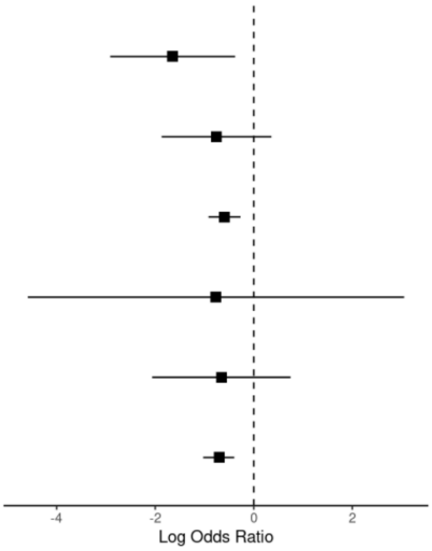

Supplementary Figure 2: Forest plots showing the within study association statistics for all genes associated with the development of prostate cancer at the suggestive significance threshold ($P < 2.6 \times 10^{-6}$). Results are from the gene-level collapsing analysis, and only the qualifying variant (QV) model with the lowest *P*-value for each gene is displayed (as indicated in parentheses). Meta-analysis odds ratios and *P*-values were determined from a Cochran–Mantel–Haenszel test across cohorts, and individual cohort odds ratios and *P*-values were calculated with Fisher's exact test (two-sided). Studies with missing data not included: full summary statistics can be found in Supplementary Data 3. All QV models defined in Supplementary Data 1. UKB, UK Biobank; MCPS, Mexico City prospective Study; 100kGP, 100,000 Genomes Project; NYBAZ Study, New York-Boston-AstraZeneca prostate cancer study; AZCT, AstraZeneca Clinical Trials.

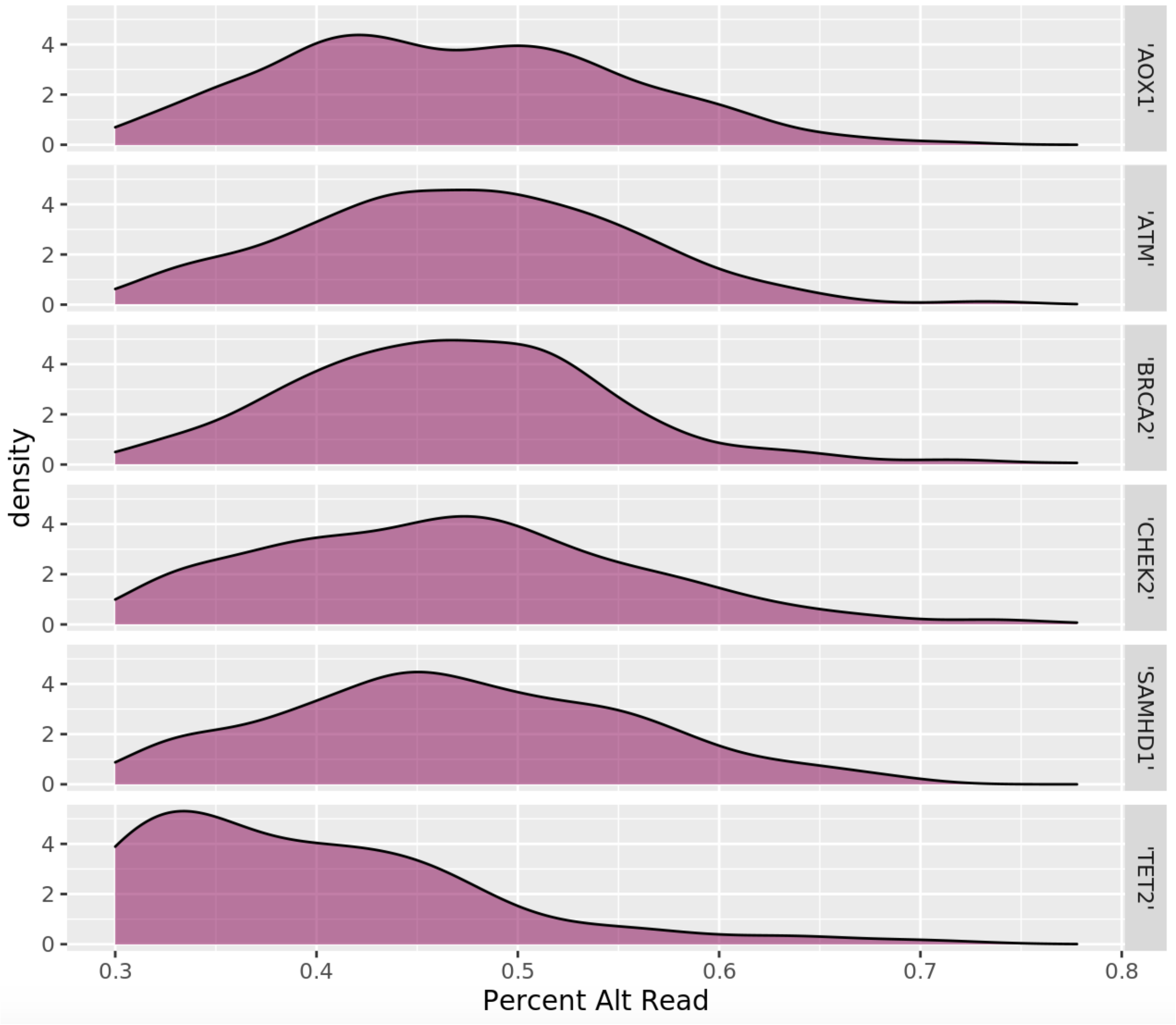

Supplementary Figure 3: Distribution of alternative (Alt) reads percentage for qualifying variants in genes associated with prostate cancer.

Gene	Odds Ratio (95% CI)	P-value
BRCA2 (ptv)	3.23 (2.65-3.90)	7.5e-29
ATM (ptv)	2.92 (2.34-3.63)	1.17e-19
CHEK2 (flexdmg)	1.69 (1.41-2.01)	2.69e-08
MSH2 (ptv)	3.38 (1.55-6.90)	0.0012
NBN (raredmgmtr)	1.92 (1.22-2.93)	0.00335
BRIP1 (raredmgmtr)	0.68 (0.46-0.97)	0.0293
PALB2 (URmtr)	1.75 (1000-2.91)	0.0415
RAD51C (flexnonsynmtr)	1.38 (0.96-1.94)	0.0656
XRCC2 (ptv)	1.68 (0.91-2.94)	0.0659
MSH6 (flexnonsynmtr)	1.15 (0.99-1.34)	0.0693
MLH1 (ptv)	2.03 (0.86-4.29)	0.0882
GEN1 (UR)	1.55 (0.87-2.60)	0.0948
BAP1 (ptv)	2.50 (0.58-8.38)	0.113
RAD51D (raredmg)	0.64 (0.35-1.08)	0.114
PMS2 (syn)	1.27 (0.93-1.71)	0.121
ATR (flexdmg)	1.10 (0.94-1.30)	0.223
BRCA1 (URmtr)	0.80 (0.55-1.14)	0.255
BARD1 (syn)	1.19 (0.85-1.62)	0.278

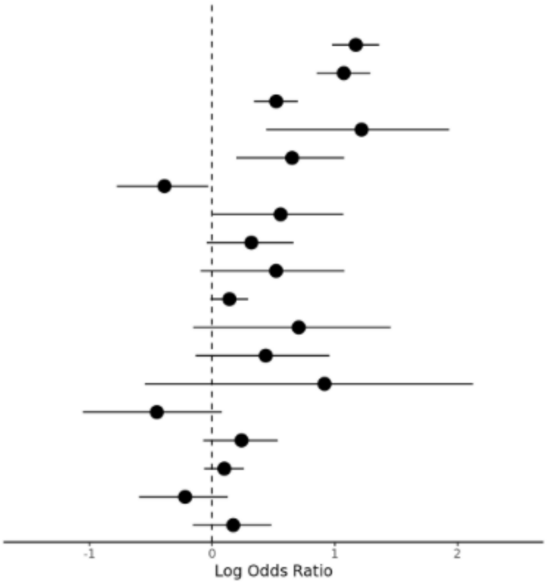

Supplementary Figure 4: Forest plots showing the meta-analysis association of DNA damage response genes with the development of prostate cancer (cases versus controls). Results are from the gene-level collapsing analysis, and only the qualifying variant (QV) model with the lowest *P*-value for each gene is displayed (as indicated in column one parentheses). Odds ratio and P-values were determined from a Cochran–Mantel–Haenszel test across cohorts. All QV models defined in Supplementary Data 1.

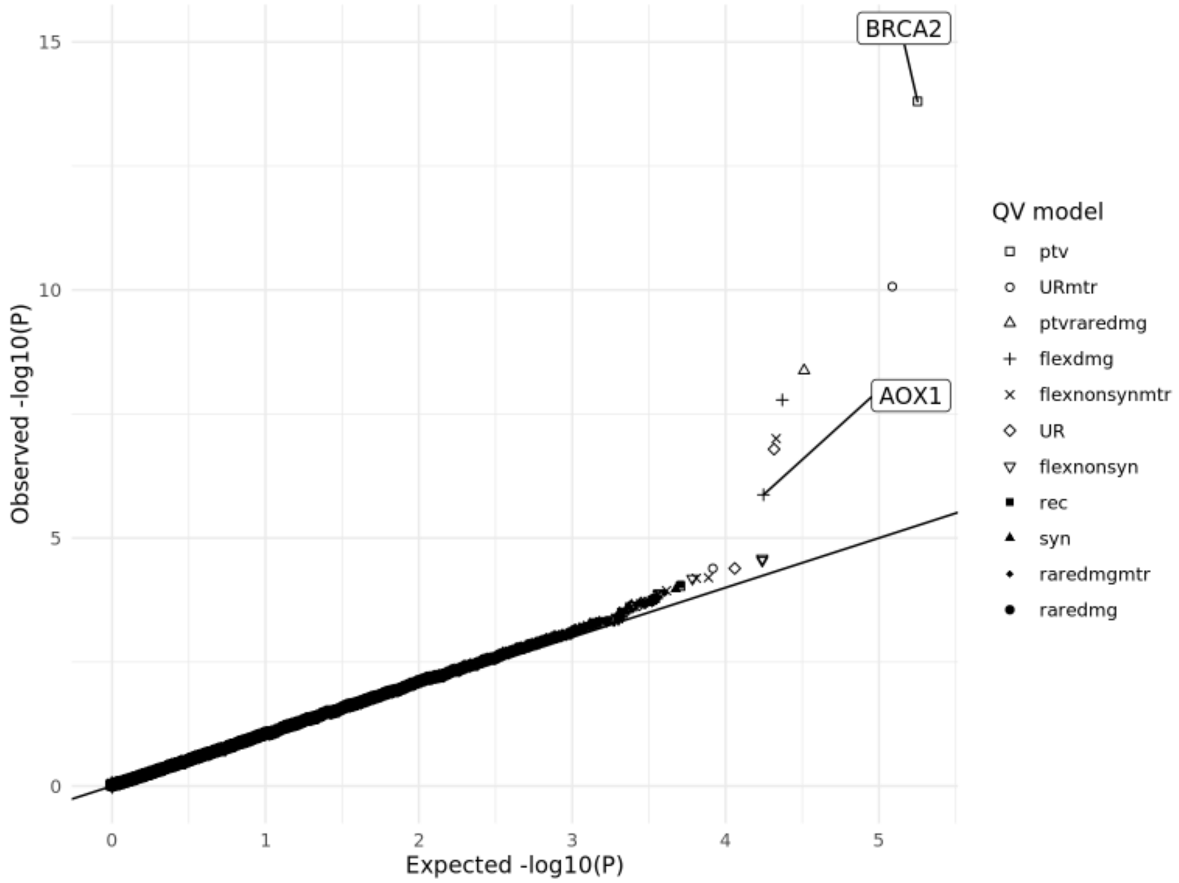

Supplementary Figure 5: QQ-plot for meta-analysis of gene-level association tests for all qualifying variant (QV) models for prostate cancer severity (aggressive prostate cancer vs non- aggressive prostate cancer). Expected *P*-values on x-axis are generated from n-1 case-control permutation. *P*-values were determined from a Cochran–Mantel–Haenszel test across cohorts. Genes which reach the suggestive significance threshold ($P < 2.6 \times 10^{-6}$) are labelled, and only the most significant QV model for each gene is labelled. All QV models defined in Supplementary Data 1.

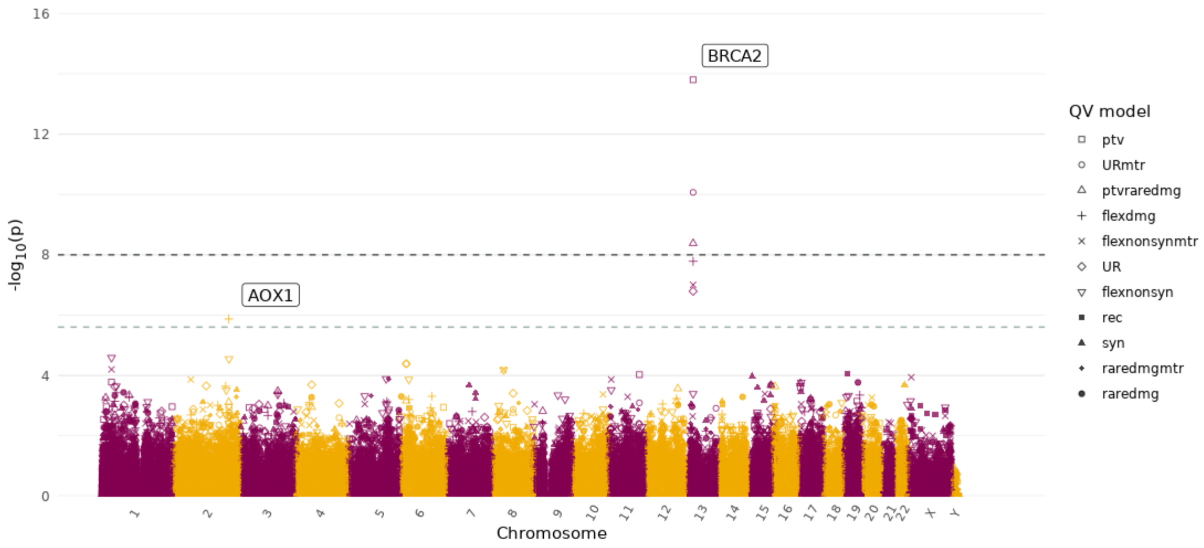

Supplementary Figure 6: Manhattan plot of all gene-level association tests with aggressive prostate cancer versus non-aggressive prostate cancer. The x-axis is the genomic position of the gene, and the y-axis is the -$\log_{10}$ transformed unadjusted *P*-values for all qualifying variant (QV) models as indicated in the legend. *P*-values were determined from a Cochran–Mantel–Haenszel test across cohorts. The light grey dashed line represents the suggestive significance threshold ($P = 2.6 \times 10^{-6}$) and the dark grey dashed line the study-wide significance threshold ($P = 1 \times 10^{-8}$). Genes which reach the suggestive significance threshold are labelled, and only the most significant QV model for each gene is labelled. All QV models defined in Supplementary Data 1.

BRCA2 (ptv)

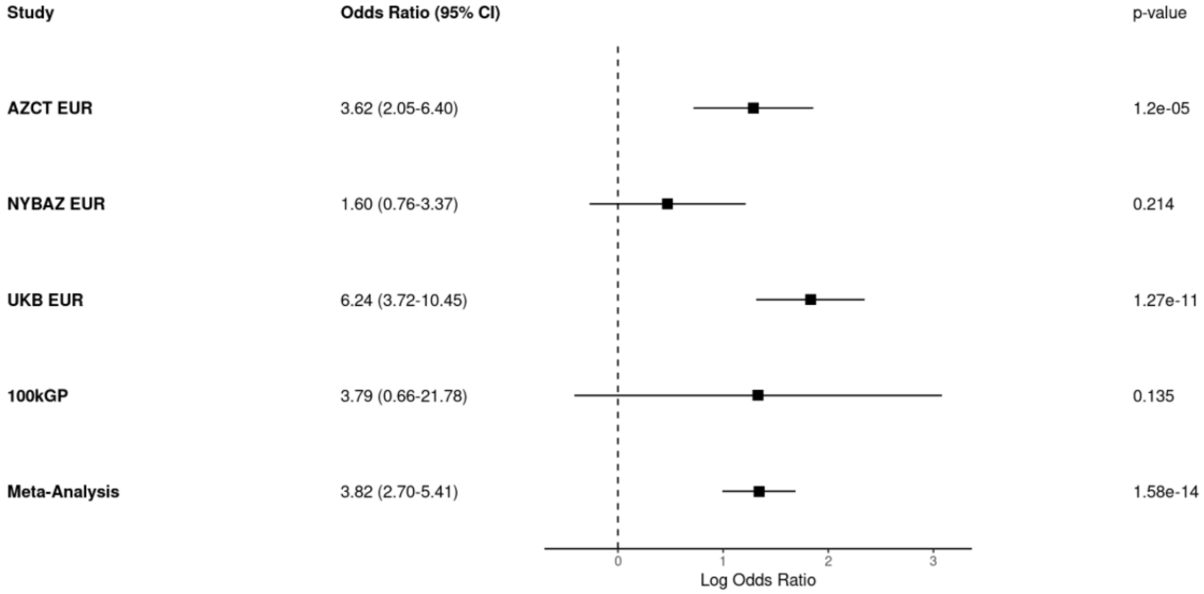

AOX1 (flexdmg)

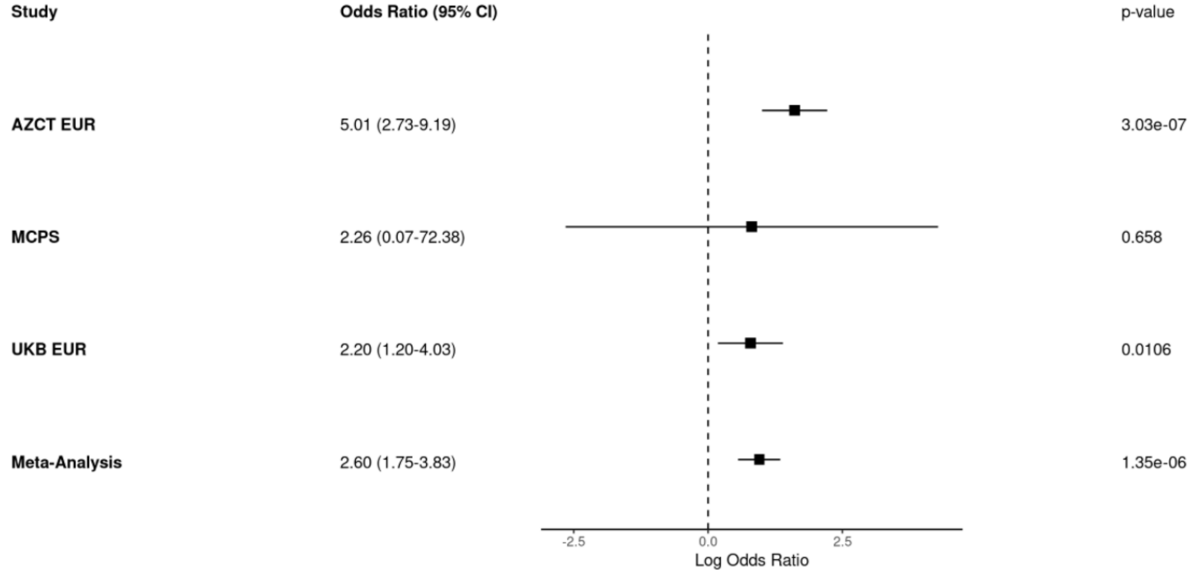

Supplementary Figure 7: Forest plots showing the within study association statistics for all genes associated with aggressive prostate cancer versus non-aggressive prostate cancer at the suggestive significance threshold ($P < 2.6\text{x}10^{-6}$). Results are from the gene-level collapsing analysis, and only the qualifying variant (QV) model with the lowest *P*-value for each gene is displayed (as indicated in parentheses). Meta-analysis odds ratios and *P*-values were determined from a Cochran–Mantel–Haenszel test across cohorts, and individual cohort odds ratios and *P*-values were calculated with Fisher's exact test (two-sided). Studies with missing data not included: full summary statistics can be found in Supplementary Data 7. All QV models defined in Supplementary Data 1.

Gene	Odds Ratio (95% CI)	P-value
BRCA2 (ptv)	3.82 (2.70-5.41)	1.58e-14
ATM (ptv)	2.23 (1.47-3.34)	9.41e-05
BARD1 (flexnonsynmtr)	1.76 (1.12-2.72)	0.0103
MSH6 (raredmg)	1.51 (1.07-2.10)	0.0143
BRCA1 (raredmg)	1.49 (1.05-2.09)	0.0217
GEN1 (ptv)	2.65 (0.98-6.71)	0.0314
RAD51C (URmtr)	6.76 (0.77-80.82)	0.0448
RAD51D (syn)	2.16 (0.88-5000)	0.0631
PALB2 (ptv)	1.96 (0.91-4.02)	0.0663
MLH1 (ptv)	3.75 (0.69-18.65)	0.0675
NBN (flexnonsyn)	1.36 (0.93-1.97)	0.0945
MSH2 (ptv)	2.54 (0.60-9.70)	0.149
CHEK2 (UR)	1.80 (0.75-4.05)	0.175
BRIP1 (raredmgmtr)	1.67 (0.69-3.78)	0.189
XRCC2 (ptv)	0.24 (0.01-1.70)	0.2
ATR (syn)	1.33 (0.80-2.15)	0.251
PMS2 (flexnonsynmtr)	0.76 (0.40-1.34)	0.359
BAP1 (syn)	1.29 (0.65-2.46)	0.406

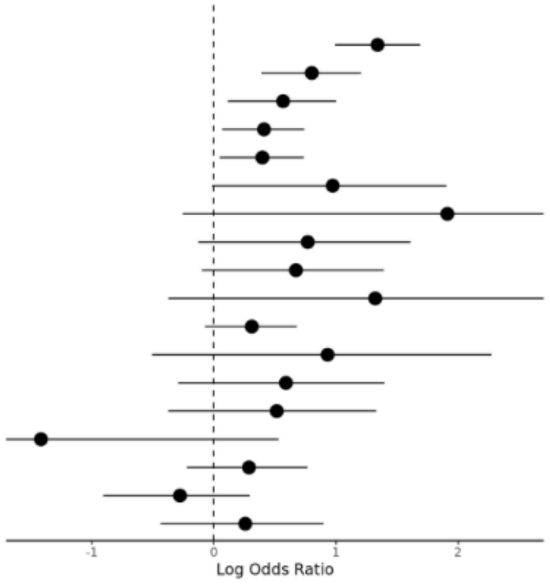

Supplementary Figure 8: Forest plots showing the meta-analysis association of DNA damage response genes with aggressive prostate cancer versus non-aggressive prostate cancer. Results are from the gene-level collapsing analysis, and only the qualifying variant (QV) model with the lowest *P*-value for each gene is displayed (as indicated in column one parentheses). Odds ratios and *P*-values were determined from a Cochran–Mantel–Haenszel test across cohorts. All QV models defined in Supplementary Data 1.

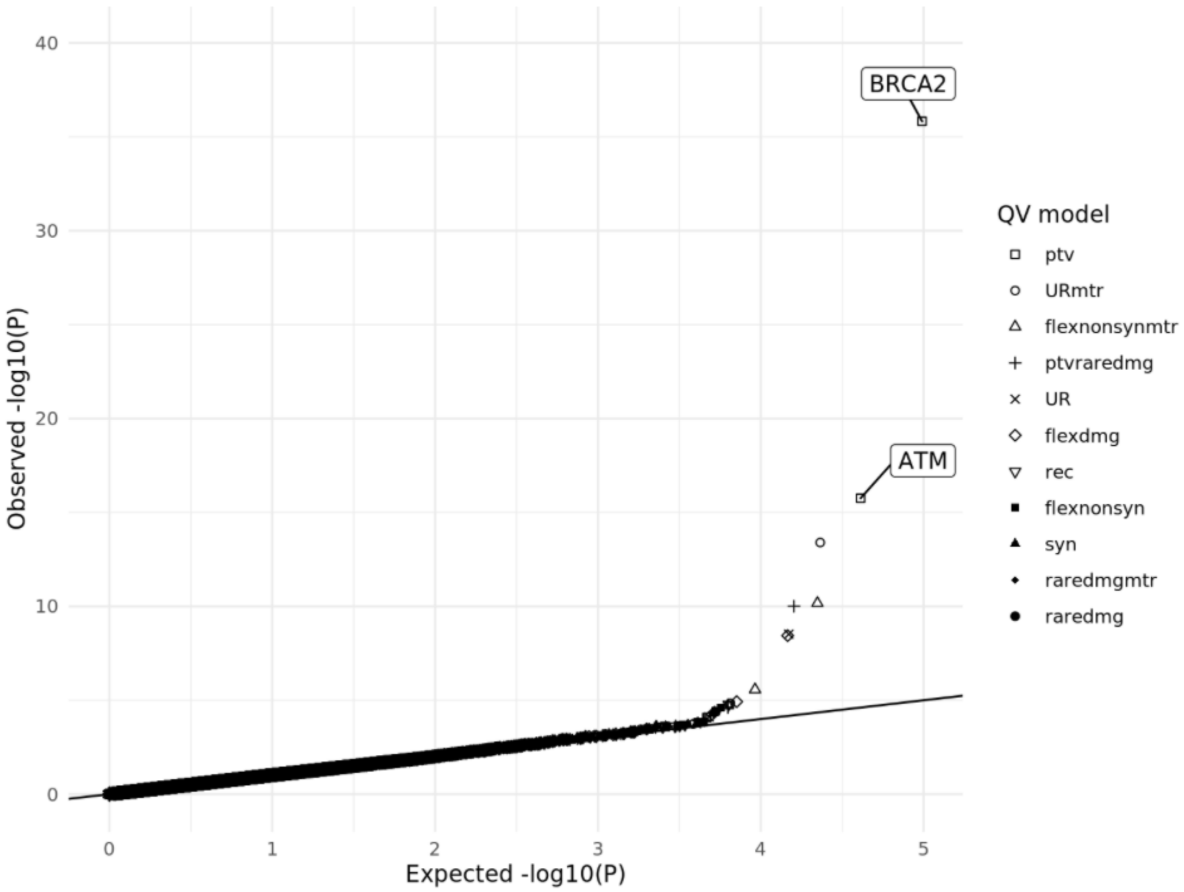

Supplementary Figure 9: QQ-plot of all meta-analyses gene-level association tests with aggressive prostate cancer versus controls. Expected *P*-values on x-axis are generated from n-1 case-control permutation. Genes which reach the suggestive significance threshold ($P < 2.6 \times 10^{-6}$) are labelled, and only the most significant qualifying variant (QV) model for each gene is labelled. *P*-values were determined from a Cochran–Mantel–Haenszel test across cohorts. All QV models defined in Supplementary Data 1.

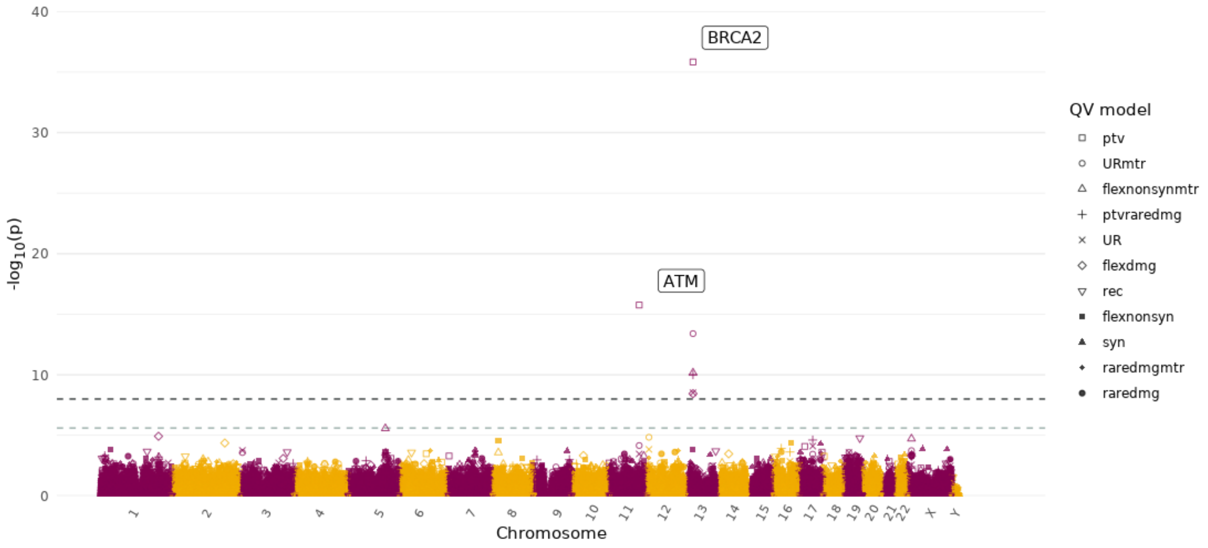

Supplementary Figure 10: Manhattan plot of all gene-level association tests with aggressive prostate cancer versus controls. The x-axis is the genomic position of the gene, and the y-axis is the $-\log_{10}$ transformed unadjusted *P*-values for all qualifying variant models as indicated in the legend. *P*-values were determined from a Cochran–Mantel–Haenszel test across cohorts. The light grey dashed line represents the suggestive significance threshold (P = 2.6x10^{-6}) and the dark grey dashed line the study-wide significance threshold (P = 1x10^{-8}). Genes which reach the suggestive significance threshold are labelled, and only the most significant qualifying variant (QV) model for each gene is labelled. All QV models defined in Supplementary Data 1.

BRCA 2 (ptv)

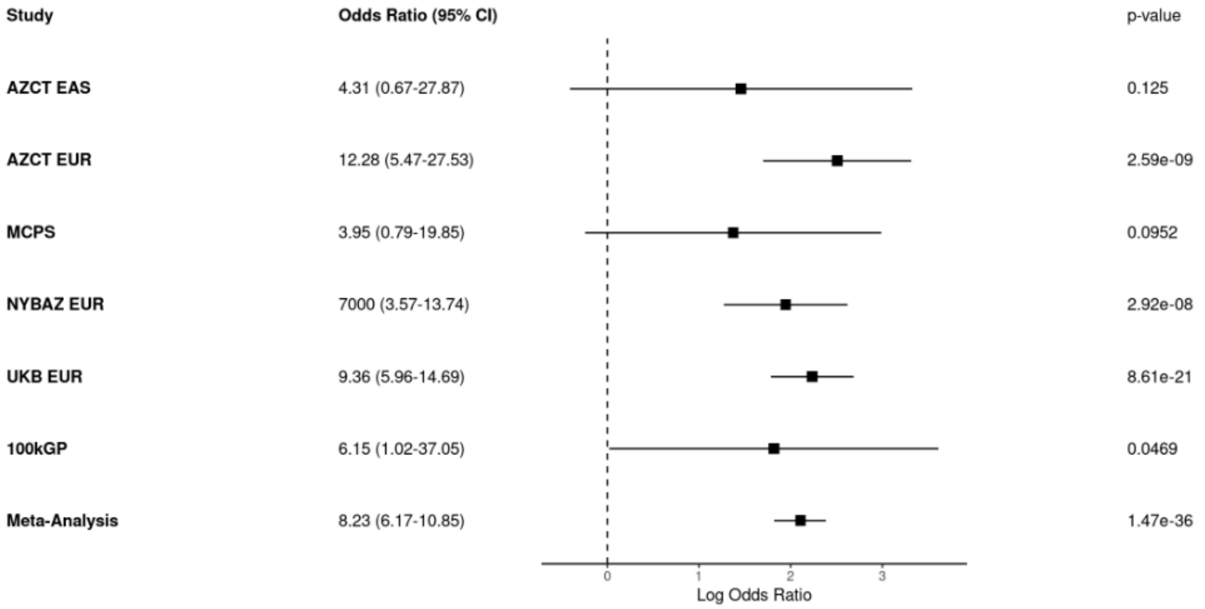

ATM (ptv)

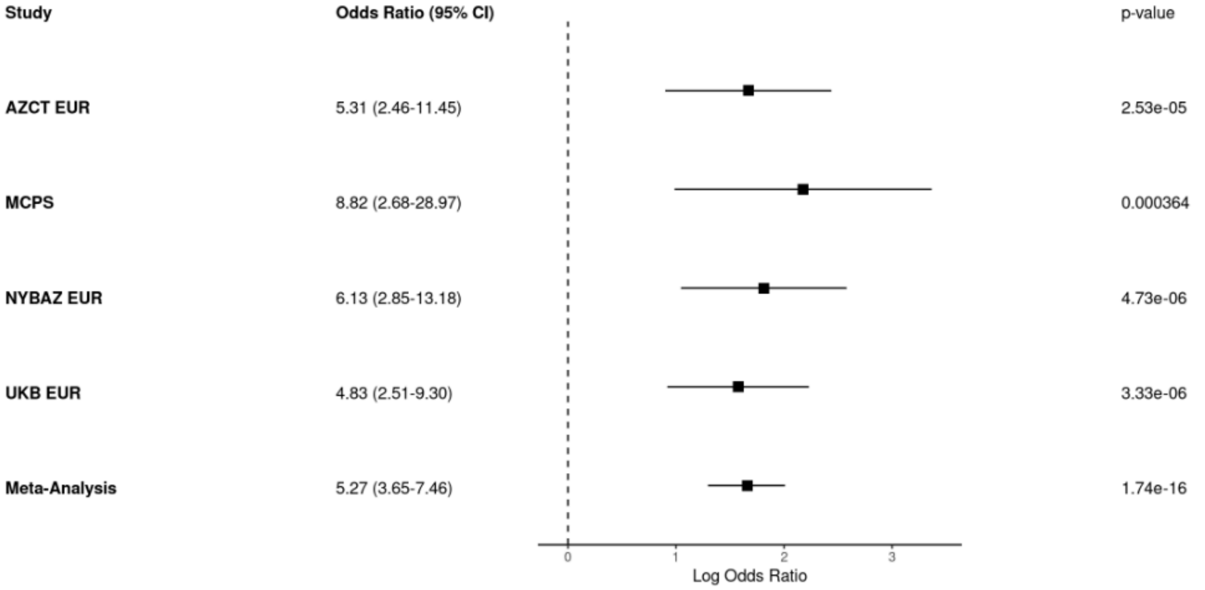

Supplementary Figure 11: Forest plots showing the within study association statistics for all genes associated with aggressive prostate cancer versus controls at the suggestive significance threshold ($P < 2.6\text{x}10^{-6}$). Results are from the gene-level collapsing analysis, and only the qualifying variant (QV) model with the lowest *P*-value for each gene is displayed (as indicated in parentheses). Meta-analysis odds ratios and *P*-values were determined from a Cochran–Mantel–Haenszel test across cohorts, and individual cohort odds ratios and *P*-values were calculated with Fisher's exact test (two-sided). Studies with missing data not included: full summary statistics can be found in Supplementary Data 11. All QV models defined in Supplementary Data 1.

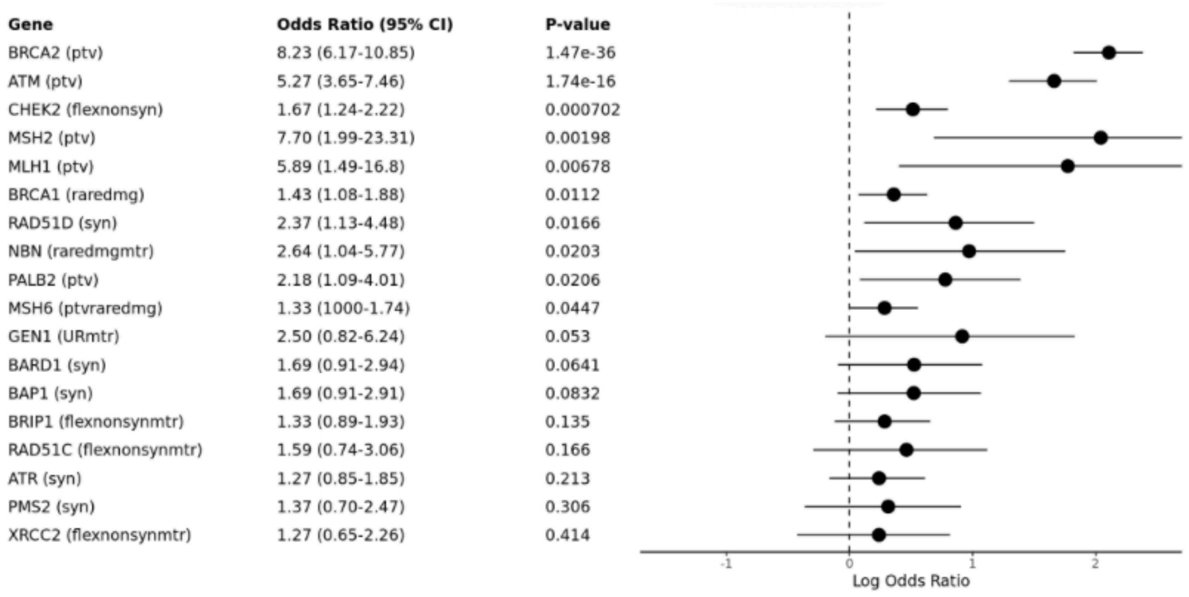

Gene	Odds Ratio (95% CI)	P-value
BRCA2 (ptv)	8.23 (6.17-10.85)	1.47e-36
ATM (ptv)	5.27 (3.65-7.46)	1.74e-16
CHEK2 (flexnonsyn)	1.67 (1.24-2.22)	0.000702
MSH2 (ptv)	7.70 (1.99-23.31)	0.00198
MLH1 (ptv)	5.89 (1.49-16.8)	0.00678
BRCA1 (raredmg)	1.43 (1.08-1.88)	0.0112
RAD51D (syn)	2.37 (1.13-4.48)	0.0166
NBN (raredmgmtr)	2.64 (1.04-5.77)	0.0203
PALB2 (ptv)	2.18 (1.09-4.01)	0.0206
MSH6 (ptvraredmg)	1.33 (1000-1.74)	0.0447
GEN1 (URmtr)	2.50 (0.82-6.24)	0.053
BARD1 (syn)	1.69 (0.91-2.94)	0.0641
BAP1 (syn)	1.69 (0.91-2.91)	0.0832
BRIP1 (flexnonsynmtr)	1.33 (0.89-1.93)	0.135
RAD51C (flexnonsynmtr)	1.59 (0.74-3.06)	0.166
ATR (syn)	1.27 (0.85-1.85)	0.213
PMS2 (syn)	1.37 (0.70-2.47)	0.306
XRCC2 (flexnonsynmtr)	1.27 (0.65-2.26)	0.414

Supplementary Figure 12: Forest plots showing the association of DNA damage response genes with aggressive prostate cancer versus controls. Results are from the gene-level collapsing analysis, and only the qualifying variant (QV) model with the lowest *P*-value for each gene is displayed (as indicated in column one parentheses). Odds ratios and *P*-values were determined from a Cochran–Mantel–Haenszel test across cohorts. All QV models defined in Supplementary Data 1.

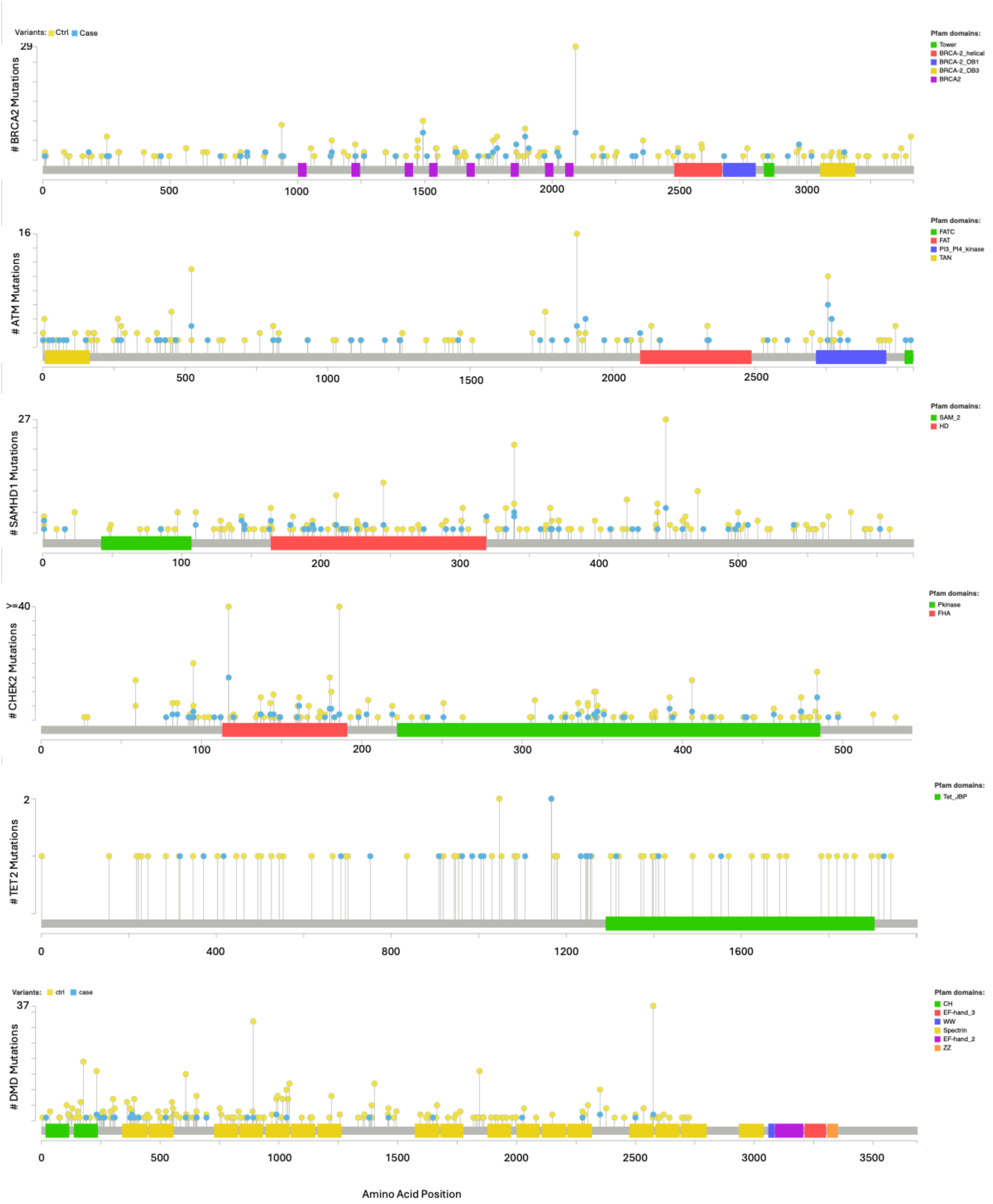

Supplementary Figure 13: Lollipop plots showing the number of qualifying variants (y-axis) for genes significantly associated with the risk of developing prostate cancer (cases versus controls). For each gene, only the most significantly associated qualifying variant (QV) model is shown (*BRCA2* = “ptv”; *ATM* = “ptv”; *SAMHD1* = “flexdmg”; *CHEK2* = “flexdmg”; *TET2* = “ptv”; *DMD* = “syn”). Variants are from UK Biobank European carriers. All QV models defined in Supplementary Data 1.

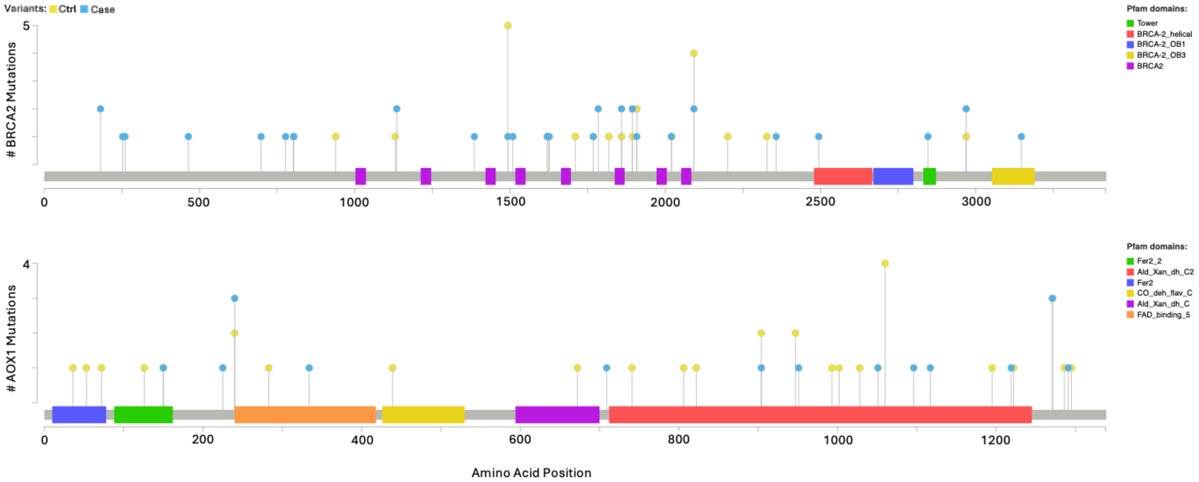

Supplementary Figure 14: Lollipop plots showing the number of qualifying variants (y-axis) for genes significantly associated with aggressive prostate cancer versus non-aggressive prostate cancer. For each gene, only the most significantly associated qualifying variant (QV) model is shown (*BRCA2* = “ptv”; *AOX1* = “flexdmg”). Variants are from UK Biobank European carriers. ctrl = non-aggressive prostate cancer, case = aggressive prostate cancer. All QV models defined in Supplementary Data 1.
