# Supplementary material for: Assessing the contribution of rare protein-coding germline variants to prostate cancer risk and severity in 37,184 cases
Source: Nat Commun. 2025 Feb 19;16:1779. doi: 10.1038/s41467-025-56944-1 (PMC11839991; doi:10.1038/s41467-025-56944-1)
Supplement: Supplementary file 2 — Description of Additional Supplementary Files [file 41467_2025_56944_MOESM2_ESM.pdf]

Description of Additional Supplementary Files

Supplementary Data 1: Criteria for qualifying variant models used in gene-level collapsing analysis.

Supplementary Data 2: Inflation factor for all gene-level QV models and all cohorts in the association tests for risk of developing prostate cancer.

Supplementary Data 3: Genes significantly associated at the suggestive level (P < 2.6x10-6) with the risk of developing prostate cancer.

Supplementary Data 4: All QV models (defined in Supplementary Table 1) are shown for genes which are significantly associated at the suggestive level (P < 2.6x10-6) with the risk of developing prostate cancer in at least one QV model.

Supplementary Data 5: DNA damage response gene associations with the risk of developing prostate cancer.

Supplementary Data 6: Inflation factor for all gene-level qualifying variant models and all cohorts in the association tests with prostate cancer severity (aggressive prostate cancer versus non-aggressive prostate cancer).

Supplementary Data 7: Genes significantly associated at the suggestive level (P < 2.6x10-6) with prostate cancer severity (aggressive prostate cancer versus non-aggressive prostate cancer).

Supplementary Data 8: All QV models (defined in Supplementary Table 1) are shown for genes which are significantly associated at the suggestive level (P < 2.6x10-6) with prostate cancer severity (aggressive prostate cancer versus non-aggressive prostate cancer) in at least one QV model.

Supplementary Data 9: DNA damage response gene associations with prostate cancer severity (aggressive prostate cancer versus non-aggressive prostate cancer).

Supplementary Data 10: Inflation factor for all gene-level qualifying variant models and all cohorts in the association tests with aggressive prostate cancer versus controls.

Supplementary Data 11: Genes significantly associated at the suggestive level (P < 2.6x10-6) with aggressive prostate cancer versus controls.

Supplementary Data 12: All QV models (defined in Supplementary Table 1) are shown for genes which are significantly associated at the suggestive level (P < 2.6x10-6) with aggressive prostate cancer versus controls in at least one QV model.

Supplementary Data 13: DNA damage response gene associations with aggressive prostate cancer versus controls.

Supplementary Data 14: Inflation factor for all ExWAS genetic models and all cohorts in association tests for the risk of developing prostate cancer.

Supplementary Data 15: Rare variants significantly associated at the study-wide level (P < 1x10-8) with prostate cancer risk.
