# Supplementary material for: Assessing the contribution of rare protein-coding germline variants to prostate cancer risk and severity in 37,184 cases
Source: Nat Commun. 2025 Feb 19;16:1779. doi: 10.1038/s41467-025-56944-1 (PMC11839991; doi:10.1038/s41467-025-56944-1)
Supplement: Supplementary file 4 — Reporting Summary [file 41467_2025_56944_MOESM4_ESM.pdf]

nature portfolio

Corresponding author(s): Dr Jonathan Mitchell
Dr Margarete Fabre

Last updated by author(s): Jan 2, 2025

Reporting Summary

Nature Portfolio wishes to improve the reproducibility of the work that we publish. This form provides structure for consistency and transparency in reporting. For further information on Nature Portfolio policies, see our Editorial Policies and the Editorial Policy Checklist.

Statistics

For all statistical analyses, confirm that the following items are present in the figure legend, table legend, main text, or Methods section.

n/a	Confirmed	
☐	☒	The exact sample size (*n*) for each experimental group/condition, given as a discrete number and unit of measurement
☐	☒	A statement on whether measurements were taken from distinct samples or whether the same sample was measured repeatedly
☐	☒	The statistical test(s) used AND whether they are one- or two-sided<br>*Only common tests should be described solely by name; describe more complex techniques in the Methods section.*
☐	☒	A description of all covariates tested
☐	☒	A description of any assumptions or corrections, such as tests of normality and adjustment for multiple comparisons
☐	☒	A full description of the statistical parameters including central tendency (e.g. means) or other basic estimates (e.g. regression coefficient) AND variation (e.g. standard deviation) or associated estimates of uncertainty (e.g. confidence intervals)
☐	☒	For null hypothesis testing, the test statistic (e.g. *F*, *t*, *r*) with confidence intervals, effect sizes, degrees of freedom and *P* value noted<br>*Give P values as exact values whenever suitable.*
☒	☐	For Bayesian analysis, information on the choice of priors and Markov chain Monte Carlo settings
☐	☒	For hierarchical and complex designs, identification of the appropriate level for tests and full reporting of outcomes
☐	☒	Estimates of effect sizes (e.g. Cohen's *d*, Pearson's *r*), indicating how they were calculated

Our web collection on statistics for biologists contains articles on many of the points above.

Software and code

Policy information about availability of computer code

Data collection	Specific software was not used in the collection of the data.
Data analysis	Publicly available software were used to perform the analyses as described in the Methods section.<br>1. Sequencing read alignment to the GRCh38 genome reference and germline variant detection: Illumina DRAGEN Bio-IT Platform Germline Pipeline v3.0.7, Isaac Genome Alignment Software, and Platypus variant caller.<br>3. Variant annotation: VEP v105 and SnpEFF v4.3.<br>2. DNA sample contamination: VerifyBAMID<br>3. Kinship: KING v2.2.3<br>4. Ancestry probability: peddy v0.4.2<br>5. Exome-wide and gene-level association studies: R v4.0.4, rvtest v2.1.0,REGENIE v2.2.4<br>6. ExWAS and gene-level meta-analyses: METAL, R v4.0.4

For manuscripts utilizing custom algorithms or software that are central to the research but not yet described in published literature, software must be made available to editors and reviewers. We strongly encourage code deposition in a community repository (e.g. GitHub). See the Nature Portfolio guidelines for submitting code & software for further information.

Data

Policy information about availability of data

All manuscripts must include a data availability statement. This statement should provide the following information, where applicable:

- Accession codes, unique identifiers, or web links for publicly available datasets
- A description of any restrictions on data availability
- For clinical datasets or third party data, please ensure that the statement adheres to our policy

For data privacy reasons individual-level data must be requested directly from each study. Individual-level UK Biobank data may be requested via application to the UK Biobank (www.ukbiobank.ac.uk/register-apply/). Individual-level MCPS data may be requested via Data and Sample Access Policy available on the study's Oxford-hosted webpage (http://www.ctsu.ox.ac.uk/research/mcps). Details on how to access the Genomics England 100K Genomes Project individual leveldata can be found at https://re-docs.genomicsengland.co.uk/pan_cancer_pub/. All FinnGen release r11 association statistics are publicly available (http://r11.finngen.fi). Summary statistics generated here for genetic association analyses are provided in the Supplementary Data.

Research involving human participants, their data, or biological material

Policy information about studies with human participants or human data. See also policy information about sex, gender (identity/presentation), and sexual orientation and race, ethnicity and racism.

Reporting on sex and gender	Sex was determined from X:Y sequencing coverage and compared to the self-reported sex.
Reporting on race, ethnicity, or other socially relevant groupings	Genetic ancestry at the continent level was determined for each participant and used to construct strata for association testing to avoid confounding.
Population characteristics	The UK Biobank (UKB) is a prospective study which recruited approximately 500,000 participants between the ages of 40 and 65 years in the United Kingdom from 2006 until 2010, of whom 46% were male. The Mexico City Prospective Study (MCPS) is a cohort of approximately 150,000 participants recruited at 35 years of age or older in Mexico City from 1998 to 2004, of whom 33% are male. The 100,000 Genomes Project recruited patients from the United Kingdom's National Health Service based on rare disease and cancer diagnoses. The New York-Boston-AstraZeneca (NYBAZ) prostate cancer study consists of prostate cancer patients from three separate cohorts: participants of the Health Professionals Follow-up Study (HPFS) and the Physicians' Health Study (PHS) who were diagnosed with prostate cancer during prospective follow-up and patients with cancer seen at the Dana-Farber Cancer Institute (DFCI) Gelb Center. The AstraZeneca clinical trial cohort contained a total of 1,445 prostate cancer patients enrolled across nine clinical trials.FinnGen is a research project encompassing 9 Finnish biobanks, and the results presented here are from approximately 445,000 participants.
Recruitment	For all cohorts, participation was voluntary. For the population cohorts (e.g. UK Biobank) especially, this introduces a bias towards the participants being healthier than the general population. We do not expect this to introduce false positives in terms of genetic variants associated with prostate cancer severity and risk.
Ethics oversight	The research presented here complies with the ethical regulations approved for each cohort. The UK Biobank (UKB) has approval from the North-West Multi-centre Research Ethics Committee (11/NW/0382), and participants provided informed consent. The Mexico City Prospective Study (MCPS) was approved by the Mexican National Council for Science and Technology, the Mexican Ministry of Health and the University of Oxford ethics committees. The 100,000 Genomes Project was approved by the National Research Ethics Committee. FinnGen study approval was obtained by the Coordinating Ethics Committee of the Hospital District of Helsinki and Uusimaa (number HUS/990/2017).

Note that full information on the approval of the study protocol must also be provided in the manuscript.

Field-specific reporting

Please select the one below that is the best fit for your research. If you are not sure, read the appropriate sections before making your selection.

☒ Life sciences ☐ Behavioural & social sciences ☐ Ecological, evolutionary & environmental sciences

For a reference copy of the document with all sections, see nature.com/documents/nr-reporting-summary-flat.pdf

Life sciences study design

All studies must disclose on these points even when the disclosure is negative.

Sample size	We did not perform any power calculations and instead use the maximum number of available samples to maximise the power for novel discovery of rare variants associated with prostate cancer risk. Total sample size across cohorts of 37,184 prostate cancer cases and 331,329 male controls.
Data exclusions	Samples were excluded based on QC criteria to mimise technical artifacts and ensure cohort harmonisation for genetic association testing (details in Methods section).
Replication	For statistically significant findings, we looked for evidence in multiple cohorts used in the meta-analysis.

Randomization	Not-applicable.
Blinding	Not-applicable.

Reporting for specific materials, systems and methods

We require information from authors about some types of materials, experimental systems and methods used in many studies. Here, indicate whether each material, system or method listed is relevant to your study. If you are not sure if a list item applies to your research, read the appropriate section before selecting a response.

Materials & experimental systems

n/a	Involved in the study
☒	☐ Antibodies
☒	☐ Eukaryotic cell lines
☒	☐ Palaeontology and archaeology
☒	☐ Animals and other organisms
☒	☐ Clinical data
☒	☐ Dual use research of concern
☒	☐ Plants

Methods

n/a	Involved in the study
☒	☐ ChIP-seq
☒	☐ Flow cytometry
☒	☐ MRI-based neuroimaging

Plants

Seed stocks	NA
Novel plant genotypes	NA
Authentication	NA
